# Factors affecting dental self-confidence and satisfaction with dental appearance among adolescents in Saudi Arabia: a cross sectional study

**DOI:** 10.1186/s12903-021-01509-z

**Published:** 2021-03-23

**Authors:** Passent Ellakany, Shaimaa M. Fouda, Maram Alghamdi, Eman Bakhurji

**Affiliations:** 1 Department of Substitutive Dental Sciences, College of Dentistry, Imam Abdulrahman Bin Faisal University, P.O. Box 1982, Dammam, 31441 Saudi Arabia; 2 Department of Preventive Dental Sciences, College of Dentistry, Imam Abdulrahman Bin Faisal University, Dammam, Saudi Arabia

**Keywords:** Psychosocial impact, PIDAQ, Dental esthetics, Adolescents, Dental self-confidence, Smile satisfaction

## Abstract

**Background:**

Dental appearance affects facial beauty, social interaction and psychological well-being particularly among adolescents. The aim of the current study was to determine factors affecting adolescent dental self-confidence and satisfaction with dental appearance in Saudi Arabia.

**Methods:**

A cross-sectional study was conducted in the Eastern Province of Saudi Arabia among 3500 students attending intermediate and high schools. Data was collected from 2637 students using the translated Arabic version of the psychosocial impact of dental esthetics questionnaire (PIDAQ) in addition to questions about smile esthetics satisfaction and demographic variables including; gender, age, school grade, and parental level of education. Statistical analysis was performed by using logistic regression to assess the effect of demographical variables on PIDAQ and its domains at 5% significance level.

**Results:**

About 80% of the participants were satisfied or somewhat satisfied with their smiles**.** Tooth alignment and tooth color were the most cited reasons for adolescents’ dissatisfaction about their smile, 34% and 33% respectively. Females and participants’ fathers’ university education figured in a statistically significant way regarding higher PIDAQ and aesthetic concerns. Females were 70%, and those with fathers’ university education were 22% more likely to have a negative psychological impact. Females expressed aesthetic concerns nearly two times more than males. Participants whose fathers possessed university education had an aesthetic concern 1.25 times more compared to those whose fathers had no school or limited school education. Females and those with mothers who had university education were less likely to have positive dental self-confidence.

**Conclusions:**

Most adolescents exhibited satisfaction with their own smiles. Smile dissatisfaction in the remaining participants was related to teeth alignment, color and shape. Females were more concerned with dental esthetics and smile satisfaction than males. Females and participants whose fathers had a university education exhibited higher psychosocial impact than males and those with or without school education. However, males showed greater self-confidence in their dental aesthetics.

## Background

Oral health evaluation methods focus mainly on dental diseases, while patients' insight about their oral well-being, including functional as well as emotional and social factors are not evaluated [1]. Patient perspective is important in determining treatment needs and to supplement traditional clinical evaluation [2]. Treatment evaluation should incorporate several health care aspects, including treatment efficiency, cost, quality of life enhancement, patient’s satisfaction and improved self-image [3].

Quality of Life (QoL) tools have been developed to meet the growing awareness of the multidimensional nature of oral health and to amend the deficiency of the normative methods. Several oral health related quality of life measuring tools are presently being used to evaluate patients’ emotions, functioning, and acceptance of their oral status [4]. Dental appearance is an essential factor of facial beauty and it can influence a person’s assumption about one's characteristics. It was suggested that good dental appearance is a prerequisite to get a prestigious job in some professions [5]. The color of teeth is a crucial factor for an esthetic smile. Discoloration of one tooth may be more obvious and adversely affects esthetics compared to generalized discoloration [6]. Additionally, tooth alignment has a great impact on dental appearance and smile satisfaction. Malocclusion is not confined to the alignment of teeth and may be a combination of skeletal and dental problems. In addition to poor oral hygiene and bad odor resulting from gingivitis, speech also might become affected by malocclusion [7]. Body image is a great concern of adolescents. It affects psychological and social adaptation as well as educational achievement [8, 9].

The Psychosocial Impact of Dental Aesthetics Questionnaire (PIDAQ) was developed to evaluate self-perception of dental aesthetics and evaluate the psychosocial influence of dental aesthetics in adolescents seeking treatment [10]. It is a validated self-rating tool that measures important aspects of the oral health-related quality of life (OHRQoL), Dental Self-confidence”, “Social Impact”, “Psychological Impact”, and “Aesthetic Concerns” [10]. “Social Impact” measures the possible problems that could be endured by an individual in social situations due to unpleasant dental appearance while “Psychological Impact” measures the feeling of sadness or inferiority in comparison to others. Aesthetic concern involves data related to the concern or disapproval caused by one’s dental appearance when an individual looks in a mirror or sees himself in photographs or videos [11].

Dentofacial esthetics has a great impact on social interaction and psychological well-being. Oral health condition, particularly satisfaction with appearance which might cause embarrassment in social contacts, affects the quality of life [8]. Thus, the aim of this study was to determine the primary causes of dental appearance dissatisfaction among adolescents in Saudi Arabia and to determine factors affecting their dental self-confidence and satisfaction with their dental appearance. The null hypothesis was that that dental appearance satisfaction is not affected by the the adolescents’ gender and educational level of the parents.

## Methods

A cross-sectional study including male and female students attending intermediate and high schools (7th to 12th grades) with an age range from 12 to 17 years old was held in the Eastern Province of Saudi Arabia. The study was conducted in three different cities: Dammam, Khobar and Jubail. Students were included in the study if they: (1) had their parents’/legal guardian’s approval to participate and (2) had no medical or psychological problems affecting their responses. The study was approved by the Research ethical committee at the College of Dentistry, Imam Abdulrahman Bin Faisal University (EA#201905). Written informed consent forms were sent to the school principals prior to data collection day and were signed and returned by the participants’ legal guardians.

Sample size was estimated for a regression model prior to the conduct of the study. The estimation was based on the following assumptions: anticipated effect size is 0.5%, with 5 predictors included in the regression model, alpha error is 5%, and study power is 80%. The study had to have 2552 respondents (https://www.danielsoper.com/statcalc/calculator.aspx?id=1). To accommodate for a possible 30% drop out and non-response, a total of 3,500 intermediate and high school students from 13 schools were invited to participate in the study. School selection was based on a random list provided by the Ministry of Education from the three different cities.

Data collection used the standardized questionnaire measuring the Psychosocial Impact of Dental Aesthetics (PIDAQ) [5]. Responses were scored as yes or no for PIDAQ items in the questionnaire.

In addition to PIDAQ items the survey included demographical variables such as gender (male, female), age (years), grade (intermediate, high school), father’s and mother’s level of education (no school, school education, or university education), besides components of dental smile esthetics satisfaction. The participants were requested to evaluate their self-satisfaction towards their smiles and to select the specific smile components which caused them to feel dissatisfied.

The survey was translated into the Arabic language by two proficient translators creating an initial draft that suits Saudi Arabia culture [1]. This initial draft was back-translated into English by both translators independently. Review of the draft was done by a committee consisting of two prosthodontists and a specialist in oral health assessment who are fluent in English. The Arabic version of the survey was tested on a sample of 20 adolescents attending intermediate and high schools in the city of Dammam (Saudi Arabia). Each adolescent answered the questionnaire independently and the time used in filling the questionnaire was recorded. The internal consistency of the PIDAQ scale in this study was acceptable (Cronbach alpha = 0.76) in comparison to the Moroccan Arabic version [1] that was compared to the original version of Klage et al. [10]. The confirmatory factor analysis showed a high comparative fit index of 0.99 and the correlation between the subscales was more than 0.7.

### Statistical analysis

IBM SPSS Statistics for Windows, Version 20.0 (Armonk, NY: IBM Corp.) was used to analyze the data. The DSC, SI, PI, AC scores were calculated by summing the participants’ responses from the corresponding question items of each domain in the questionnaire. Additionally, the total PIDAQ score was calculated from the sum scores of the subdivisions AC, PI, SI, and the reversed scores of the positive domain DSC. The scores of all domains were then dichotomized based on the median into low and high impact. The low PIDAQ, PI, SI, AC scores reflected no negative psychosocial impact, while the high PIDAQ scores reflected high negative psychosocial impact. The reverse is true (low means negative and high means positive) for the DSC since it is a positive measure. Frequencies (N) and percentages (%) were calculated for categorical variables while mean (M) and standard deviation (SD) were calculated for continuous variables. Simple (unadjusted) and multiple (adjusted) logistic regressions were performed to assess the effect of demographical variables on PIDAQ and other domains. All analyses were performed at 5% significance level.

## Results

### Response rate and participants’ characteristics

The survey was distributed to 3500 students. Of the distributed surveys, 2637 were completed and returned for a response rate of 75.34%. Table 1 presents the demographic distribution of the study participants. About two thirds of the study respondents were intermediate school students (63%), and most were females (62%) with a mean age of 14.52 ± 1.78 years old. Based on parents’ level of education, a higher percentage of participants' fathers had university level of education (50.6%) compared to their mothers (41.7%).

**Table 1:** Characteristics of study participants (N = 2637)

Study variables	N (%)
Grade	
Intermediate school	1662 (63)
High school	975 (37)
Gender	
Male	996 (37.8)
Female	1641 (62.2)
Father’s education level (N = 2343)	
No/school education	1157 (49.4)
University education	1186 (50.6)
Mother’s education level (N = 2391)	
No/school education	1395 (58.3)
University education	996 (41.7)
Age (years)	Mean ± SD
	14.52 ± 1.78

### Distribution of participants’ smile satisfaction according to PIDAQ questions

Most of the participants were satisfied (37.4%) or somewhat satisfied (42.5%) with their smiles compared to only 20% who were not satisfied with their smiles. A majority of the participants did not hide their smiles (71%), were not aware of other people’s views of their smile (75.6%), and their smile did not make them self-conscious in the presence of their family or friends (81.4%). However, 68% of them were not comfortable showing their teeth when smiling, 57% did not like to display their teeth in the mirror, photographs or videos, and 93% wished their teeth look better. On the other hand, 46% of respondents were happy with their smiles and 38% reported that their teeth were not the reason for their smile dissatisfaction (Table 2).

**Table 2:** Distribution of study participants’ responses to PIDAQ questions (N = 2637)

Questions				
1. How much are you satisfied with your smile?	Satisfied	Somewhat satisfied		Not satisfied
	987 (37.4)	1121 (42.5)		529 (20.1)
	Yes			No
2. Have you noticed that you hide your teeth when you smile?	775 (29)			1872 (71)
3. Are you comfortable with showing your teeth while smiling?	1805 (68.4)			832 (31.6)
4. Do you like your teeth display in mirrors, photographs and videos?	1500 (56.9)			1137 (43.1)
5. Have you a perceived notion about other people's views of your smile?	643 (24.4)			1994 (75.6)
6. Does your smile make you self-conscious in the presence of family and friends?	490 (18.6)			2147 (81.4)
7. Do you wish that your teeth looked better?	2446 (92.8)			191 (7.2)
	Yes		No	Happy with my smile
8. Are your teeth the reason for your dissatisfaction with your looks?	417 (15.8)		999 (37.9)	1221 (46.3)

### Reasons for participants’ dissatisfaction about their smile appearance

**Fig. 1:**
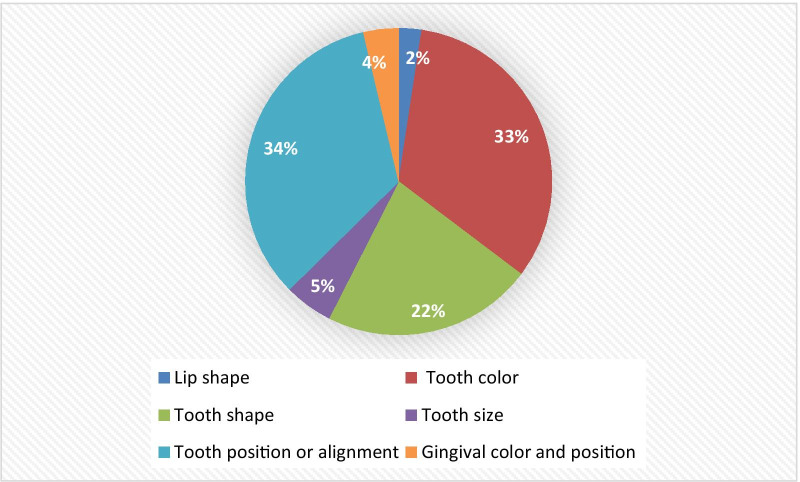
Reasons for participant’s dissatisfaction about their smile appearance (%) (N = 1022)

Regarding the reasons for smile dissatisfaction among the study participants, Fig. 1 presents different predetermined reasons for smile dissatisfaction. Tooth alignment and tooth color were the most cited reasons for adolescents’ dissatisfaction about their smile, 34% and 33%, respectively, while 22% did not like the shape of their teeth. On the other hand, tooth size (5%), tooth position, gingival color (4%), and lip shape (2%) were the least selected reasons for smile dissatisfaction.

### Factors affecting dental self-confidence and satisfaction with dental appearance

Univariate and multiple logistic regression models predicting high psychosocial impact of dental aesthetics (PIDAQ), low DSC, high SI, PI, and AC are shown in Table 3. Gender was the main predictor in most models followed by father’s level of education. Some models showed that one’s mother’s level of education was of importance as well.

**Table 3:** Logistic regression models predicting PIDAQ and its various components by demographical variables (N = 2637)

	OR (95% CI)									
	PIDAQ		DSC		SI		PI		AC	
	Unadjusted	Adjusted	Unadjusted	Adjusted	Unadjusted	Adjusted	Unadjusted	Adjusted	Unadjusted	Adjusted
*Gender*										
Males (ref)	–	–	–	–	–	–	–	–	–	–
Females	1.68 (1.42–1.97)^a^	1.74 (1.45–2.10)^a^	0.71 (0.61–0.84)	0.69 (0.58–0.83)^a^	1.12 (0.95–1.3)	1.12 (0.94–1.34)	1.58 (1.34–1.86)	1.70 (1.41–2.1)^a^	1.94 (1.65–2.27)^a^	1.93 (1.61–2.31)^a^
*Grade*										
Intermediate School (ref)	–	–	–	–	–	–	–	–	–	–
High School	0.97 (0.83–1.14)	1.1 (0.81–1.48)	1.1 (0.94–1.3)	0.95 (0.71–1.28)	0.87 (0.74–1.02)	0.97 (0.72–1.31)	0.91 (0.77–10.7)	1.07 (0.79–1.45)	1.33 (1.1–1.5)^a^	1.10 (0.81–1.48)
Age (years)	1.03 (0.99–1.03)	1.06 (0.97–1.15)	0.95 (0.91–0.99)	0.94 (0.87–1.02)	1.04 (0.99–10.9)	1.04 (0.96–1.12)	1.05 (1.01–1.10)	1.1 (0.99–1.17)	0.94 (0.90–0.99)^a^	0.98 (0.91–1.07)
*Father’s education level*										
No/School (ref)	–	–	–	–	–	–	–	–	–	–
University	1.39 (1.18–1.64)^a^	1.34 (1.11–1.61)^a^	0.78 (0.66–0.92)	0.88 (0.73–1.05)	1.14 (0.97–1.35)	1.1 (0.88–1.26)	1.31 (1.11–1.55)	1.22 (1.01–1.47)^a^	1.2 (0.99–1.39)	1.25 (1.04–1.60)^a^
*Mother’s education level*										
No/School (ref)	–	–	–	–	–	–	–	–	–	–
University	1.27 (1.08–1.50)*	1.13 (0.94–1.36)	0.72 (0.61–0.85)	0.76 (0.63–0.91)^a^	1.24 (1.05–1.45)^a^	1.18 (0.98–1.41)	1.26 (1.07–1.49)	1.14 (0.94–1.37)	1.08 (0.91–1.27)	1.08 (0.90–1.30)

^a^Statistically significant

Females were statistically significantly more likely to have higher psychosocial impact of dental aesthetics (unadjusted OR 1.68 95% CI 1.42, 1.97, adjusted OR 1.74 95% CI 1.45–2.10). Additionally, participants fathers’ university education was statistically significantly associated with higher odds of having a psychosocial impact of dental esthetics (PIDAQ) (unadjusted OR 1.39 95% CI 1.18, 1.64, adjusted OR 1.34 95% CI 1.11, 1.61). Grade, age or mothers’ education level did not significantly affect the PIDAQ scores of the participants. Similarly, females were 70% and those with fathers having university education were 22% more likely to have negative psychological impact (PI) in the adjusted multivariate model (95% CI 1.41, 2.1 and 1.01, 1.47, respectively).

When examining aesthetic concern (AC), it was found that females had 1.93 times the odds of having an aesthetic concern (AC) compared to males (95% CI 1.61, 2.31). Participants whose fathers possessed university education had 1.25 times the odds of having an aesthetic concern in comparison to those whose fathers had no school or limited school education (95% CI 1.04, 1.60).

In the DSC adjusted logistic model, gender and participants mothers’ education were the only statistically significant predictors in the model. Females and those with mothers’ having university education were less likely to have positive DSC (adjusted OR 0.69 95% CI 0.58, 0.83 and OR 0.76 95%CI 0.63, 0.91, respectively). Additionally, those with mothers having university education were 25% statistically significantly more likely to have higher social impact (SI) in the unadjusted logistic model (95% CI 1.05, 1.45). Although the odds ratio for the mothers’ education level does not change a lot in the adjusted model, it becomes non-significant (OR 1.18 95% CI 0.98, 1.41).

## Discussion

Patient satisfaction and aesthetic concern are important factors that must be considered for a successful dental treatment [12]. The present study determined factors affecting adolescent dental self-confidence and satisfaction with dental appearance in Saudi Arabia. The results showed that tooth color and alignment, gender and parents’ educational level affected their dental self-confidence and smile satisfaction. Thus, the null hypothesis was rejected.

The results of the current study showed that most students (80%) were satisfied or somewhat satisfied with their smiles. However, the main reasons for smile dissatisfaction were related to teeth alignment, color and shape respectively. Similarly, previous studies showed that patients feel better and safer when they are pleased with the alignment and shape of their teeth, and that teeth crowding results in negative psychosocial effects [12–15]. Malocclusion affects facial appeal, thus influencing self and social perception of adolescents [15], due to the association between appearance and social status and acceptability [16]. In line with our results, several studies found that patients’ satisfaction with their dental appearance was affected by tooth color [8, 12–14, 17, 18]., Tooth discoloration could decrease a patient’s personal satisfaction and adversely affects his emotional state, Therefore, some patients seek cosmetic treatments including tooth whitening [17]. Contrary to our findings, Höfel et al. [19] reported that neither tooth color, nor dental appearance were correlated to perceptions of facial attractiveness. This variation in the results might be due to differences between the study design regarding age and educational level of the participants. [19].

Our results revealed that females felt a higher psychosocial impact of dental aesthetics and aesthetic concern (AC) compared to males, and lower dental self-confidence (DSC). These results are in agreement with previous reports that observed less self-confidence in girls compared to boys [20, 21]. Also in agreement, previous studies found higher dental concern and oral demands with females than males, who are more comfortable with their dental appearance [22, 23]. This might be related to their social life style that makes them less concerned with their appearance than females [22, 23]. In addition, psychosocial factors are the main motivation that make females require esthetic treatment, therefore these factors had significantly higher psychological and social impact than males [23, 24]. Other studies didn’t find significant differences in the psychosocial impact of dental aesthetics between males and females [25, 26]. In contrast, Chen et al. [27] found that males, compared to females, showed more adverse aesthetic attitude and dental self-confidence when anterior teeth were missing, and higher improvement of the social impact, aesthetic attitude and dental self-confidence after implantation [27]. This can be explained by the opinion that males are generally less stable psychologically and live a more stressful social life than females [27]. Afroz et al. [5] found that Indian men more concerned about their smile than women were, and women were more satisfied with their dental aesthetics. The authors suggested that changes in society and the impact of marketing made men as concerned as women with their beauty and their physical appearance [5, 25]. The diversity between these findings and the present study could have resulted from differences in age of the participants and in the study methods [22] or due to the ethnic and cultural differences between the studied populations [28].

Obvious malocclusion, tooth color, and being a female are among the factors that increase aesthetic concern (AC) [13]. These findings are in line with the present study which reported a higher AC among females in comparison to males, as well as showing the effect of tooth alignment and color on patients’ dissatisfaction with their smile.

University education of participants' fathers was significantly associated with higher psychosocial impact of dental aesthetics, high psychological impact (PI) and aesthetic concern (AC). Participants whose mothers had a university education expressed less positive dental self-confidence (DSC) and high social impact (SI). In line with the present results, a previous study suggested that individuals with higher education are aware of the effect of dental esthetics on social acceptance [27, 29]. However, Akarslan et al. [18] correlated decreased dissatisfaction with dental aesthetics and an increase in educational level.

Romero et al. [30] found that participants with university degrees presented higher scores in self-confidence than participants with just school education. This might be attributed to the increase in maturity with age and knowledge, which is in agreement with our results as the participants were from intermediate and high schools. But their parents’ education might have raised the participants’ needs and aesthetic expectations and decreased their smile satisfaction because participants are looking to reach the best esthetic outcome like their parental role models [31].

There is a strong correlation between dental treatment needs, especially esthetic treatments, and psychological satisfaction with dental appearance, that is affected by poor tooth color and alignment [13, 14]. This agrees with our results which showed that causes of smile dissatisfaction were related mainly to improper tooth alignment, color and shape. Understanding the treatment needs of adolescents would have an important clinical significance particularly when planning cosmetic dental treatment satisfying the patient’s needs and expectations [13].

The strengths of this study include the high response rate of participants from different areas of the Eastern Province of Saudi Arabia. Hence, the results are representative of the adolescent population in the area of study. However, this study was limited to testing satisfaction with dental appearance among adolescents. Therefore, the results of this study do not represent older age groups and cannot be generalized to the whole population.

Further long-term longitudinal studies are required to evaluate the effect of age, level of education, income, social status, and different conditions (physical and psychological) on satisfaction with dental appearance and the psychosocial impact of dental aesthetics.

## Conclusions

Most adolescents exhibited satisfaction with their own smiles while those were dissatisfied felt that way mainly because of their teeth alignment, color and shape. Females exhibited higher psychosocial impact than males, while males showed greater self-confidence in their dental aesthetics. Females showed more aesthetic concern than male participants in addition to the higher concern of participants whose fathers had a university education in comparison to those with lower or no education. Also, participants whose parents possessed a university education exhibited higher psychosocial impact and esthetic demands than those whose parents did not possess higher levels of education.

Dentists should pay more attention to these traits and to their significance when treating patients.

## Data Availability

All data used and analyzed during the current study are available from the corresponding author on reasonable request.

## Appendix

### Questions of the esthetic survey questionnaire

Demographic information

1. Grade

a. Intermediate school

b. High school

2. Gender

a. Male

b. Female

3. Age

4. Father’s level of education

a. Illiterate

b. Finished primary school

c. Finished intermediate school

d. Finished high school

e. College or university education

f. Father is not around

g. I do not know

5. Mother’s level of education

a. Illiterate

b. Finished primary school

c. Finished intermediate school

d. Finished high school

e. College or university education

f. I do not know

Please select all answers that apply:

6. How much are you satisfied with your smile?

a. Highly satisfied

b. Satisfied

c. Not satisfied

7. What according to you is not satisfactory about your smile? (More than one can be chosen)

a. Lip shape

b. Tooth colour

c. Tooth shape

d. Tooth size

e. Tooth position or arrangement

f. Gingival color and position

8. Have you noticed that you hide your teeth when u smile?

Yes/No

9. Are you comfortable with showing your teeth while smiling?

Yes/No

10. Do you like your teeth display in mirror, photographs and videos?

Yes/No

11. Have you perceived notion about other people’s views of your smile?

Yes/No

12. Does your smile make you conscious in presence of opposite sex?

Yes/No

13. Do you wish that your teeth looked better?

Yes/No

14. Is your teeth the reason of your dissatisfaction with your looks?

Yes/No.

## Abbreviations

PIDAQ: Psychosocial Impact of Dental Aesthetics Questionnaire
PI: Psychological Impact
DSC: Dental self-confidence
QoL: Quality of life
OHRQoL: Oral health-related quality of life
SI: Social impact
AC: Aesthetic concern
EA: Ethical approval
SD: Standard deviation
N: Frequency
M: Mean
%: Percentage
OR: Odds ratio
CI: Confidence interval
